# The Clinical Relevance of Diabetes Distress versus Major Depression in Type 2 Diabetes: A Latent Class Analysis from the Fremantle Diabetes Study Phase II

**DOI:** 10.3390/jcm12247722

**Published:** 2023-12-16

**Authors:** Wendy A. Davis, David G. Bruce, Timothy M. E. Davis, Sergio E. Starkstein

**Affiliations:** Medical School, The University of Western Australia, Fremantle Hospital, Alma Street, Fremantle, WA 6160, Australia; wendy.davis@uwa.edu.au (W.A.D.); david.bruce@uwa.edu.au (D.G.B.); sergio.starkstein@uwa.edu.au (S.E.S.)

**Keywords:** type 2 diabetes, diabetes distress, depression, anxiety

## Abstract

Background: The nosological position and clinical relevance of the concept of diabetes distress (DD) are uncertain. The aim of this study was to use latent class analysis (LCA) to categorise classes of people with type 2 diabetes and to compare their characteristics. Methods: Data from 662 participants in the longitudinal observational Fremantle Diabetes Study Phase II were analysed. LCA identified latent subgroups based on individual responses to the Patient Health Questionnaire-9, the Generalised Anxiety Disorder Scale, and the 5-item Problem Areas in Diabetes Scale. Results: Four classes were identified: Class 1 (65.7%, no symptoms), Class 2 (14.0%, DD), Class 3 (12.6%, subsyndromal depression (SSD)), and Class 4 (7.6%, major depression (MD)). Multinomial regression analysis with Class 1 as reference showed significant associations between the DD class and Southern European and Asian ethnic background, HbA_1c_, and BMI. The SSD class was significantly associated with HbA_1c_, cerebrovascular disease, and coronary heart disease (CHD). The MD class had significant associations with age (inversely), Southern European ethnic background, HbA_1c_, BMI, and CHD. In conclusion, LCA identified a pure DD group comprising 14.0% of participants. The only variable uniquely associated with the DD class was Asian ethnic background. Conclusion: Although identification of DD may have some utility in assessing the psychological wellbeing of individuals with type 2 diabetes, it adds little to the assessment of depressive disorder and its significant clinical sequalae.

## 1. Introduction

Diabetes distress (DD) is a psychological construct used increasingly in the clinical assessment of individuals with diabetes. It has been variously conceptualised as the emotional burden and worry from living with a severe and chronic disorder [1], the psychological adjustment to challenges faced by people with diabetes [2,3], concern about disease management and access to care [4], a negative emotional response to the demands of diabetes [5,6], inadequate adjustment in people with diabetes and depressive symptoms [7], and an understandable emotional response to unpleasant stressors and the burden of diagnosis and treatment [8]. DD phenomenology is heterogeneous and includes hopelessness, fears, frustration, worry, anxiety, poor adjustment, anger, discouragement, depression, discomfort, concern, guilt, anxiety, and loneliness [8,9,10]. DD is diagnosed from scores above cut-off points on dedicated rating scales but not on standardised criteria [11], reflecting a lack of uniform conceptualisation of DD as a clinical condition [1]. The early concept of DD was that of the worries, concerns, and fears among individuals struggling with a progressive and demanding clinical disease [5], and the benefit of assessing DD in clinical practice was to signal limitations in diabetes self-management [5]. Despite an increasing literature and wide acceptance of DD, its nosological and conceptual position is unclear. Some studies consider DD as a non-pathological emotional change independent of anxiety and depression [12] and characterised by stress, fear, emotional burden, and chronic worrying [1,13], whereas other researchers have found a significant association between DD and psychopathology [12]. The overlap between DD and depression has been noted in several studies [12].

Given the high prevalence and clinical impact of depression in type 2 diabetes [14], a relevant issue is the strength of the phenomenological and nosological relationship between DD and depression. DD has been considered to be not only phenomenologically different from depression but also a causative factor in depression complicating type 2 diabetes [2,3]. Other authors consider DD to differ from depression given the former is regarded as a type of emotional distress stemming from living with the burden of a chronic illness [2]. Still others consider that elevated depression scores may not indicate depression but rather DD [12], and that depression, anxiety, and DD are independent but partially overlapping constructs [15]. Moreover, DD is described as conceptually different from depression in that it primarily captures fears, worries, and frustrations associated with diabetes self-management [7] as distinct from a depressive mood disorder [16]. Aikens has suggested that, whereas depression may impair self-management behaviours, DD impacts treatment adherence and optimal glycaemic control [17].

In relation to clinical differences between DD and depression, it has been suggested that DD but not depression implies aetiology, that only DD is content-related, and that, unlike depression, DD does not assume psychopathology [9]. Arguing against formulating DD and depression as independent constructs, the 20-item Problem Areas in Diabetes scale (PAID-20) has been recommended for diagnosing subtypes of depression and, conversely, it has been proposed that depression scales are useful in screening for DD [10]. Indeed, several PAID-20 items are related to generic states of emotional distress such as depression [10]. Based on these reports, it can be concluded that the conceptual and phenomenological bases of DD are heterogeneous, and that the validity of this construct, as well as its independence from depression and anxiety, must be clarified. It has been suggested that the statistical technique of latent class analysis (LCA) of data from large samples should be used to examine the nosological status of DD [18].

Given this background, we have used data from community-based participants with type 2 diabetes from the Fremantle Diabetes Study Phase II (FDS2) and LCA to identify a DD class and its potential overlap with depressive and anxiety symptoms. As an extension of a previous LCA study of depression from our group [19], our hypotheses were (i) given that DD diagnosis is based on psychological symptom rating scales, DD is a psychological class independent of depression and anxiety, (ii) based on the literature [20], DD is associated with suboptimal adherence to diabetes medications and recommended self-monitoring of blood glucose (SMBG) relative to anxiety and depression, and (iii) given the conceptual and empiric overlap between DD, anxiety, and depression, there is a class of people with type 2 diabetes and major multifactorial psychopathology.

## 2. Materials and Methods

### 2.1. Study Site, Participants, and Approvals

The FDS2 is a longitudinal observational study of known diabetes conducted in a zip-code-defined area surrounding the port of Fremantle in the state of Western Australia [21]. Details of recruitment, sample characteristics including classification of diabetes type, and non-recruited patients have been published [21]. Briefly, between 2008 and 2011, 4639 people with known diabetes were identified in the local population of 157,000, of whom 1668 (36%) were recruited to the FDS2 in addition to 64 surviving FDS Phase I participants who had moved out of the study area. Of these 1732 participants, 1551 had clinically defined type 2 diabetes. The Human Research Ethics Committee of the Southern Metropolitan Area Health Service approved the FDS2. All participants gave written informed consent. Participants in the FDS2 were scheduled for comprehensive face-to-face assessments at baseline and biennially to Year 6 [21]. The present cross-sectional sub-study included a subset of 662 with type 2 diabetes who attended the Year 6 assessment, excluding those found to have maturity-onset diabetes of the young or latent autoimmune diabetes of adults after initial categorisation of diabetes type.

### 2.2. Clinical and Psychiatric Assessment

At baseline and each biennial face-to-face review, detailed standardised questionnaire responses and clinical and laboratory data were collected [21]. Questionnaires covered socioeconomic, demographic, and lifestyle aspects, healthcare utilisation, all medical conditions, and medication use. Ethnic background was based on self-selection, country/countries of birth and parents’ birth, country/countries of grandparents’ birth, and language(s) spoken at home [22]. A physical examination was conducted by trained nurses according to a standard protocol. Biochemical tests were performed on fasting samples using validated automated methods in a single nationally accredited laboratory. Chronic complications were identified using standard definitions [21]. Self-monitoring of blood glucose (SMBG) was ascertained by interview, as was compliance with diabetes medication. In addition to body mass index (BMI), the A Body Shape Index (ABSI) was calculated as m^11/6^/kg^2/3^ [23].

All participants were asked to complete the PHQ-9 [24], a brief and accurate assessment of depression validated in people with diabetes [25]. The PHQ-9 is a dual-purpose instrument that can establish depressive disorder diagnoses as well as ratings of depressive symptom severity [24]. Using a PHQ-9 cut-off point rather than the diagnostic algorithm may increase the frequency of false-positive cases of depression [26]. Therefore, as suggested by the PHQ-9 authors [26], we used the instrument’s diagnostic algorithm for diagnosing major depression, specifically when a depressed mood or loss of interest/anhedonia were present more than half of the days, and at least five of the nine symptoms were present during the same period [27]. Subsyndromal depression (SSD) was diagnosed whenever two to four symptoms (including depressed mood or loss of interest/anhedonia) were present for more than half of the days.

To examine the frequency and severity of anxiety in FDS2, we developed and validated the Generalised Anxiety Disorder Scale (GADS) [8,19], which includes items corresponding to all nine Diagnostic and Statistical Manual of Mental Disorders Fourth/Fifth Edition (DSM-IV/V) criteria for GAD. Based on the PHQ-9, GADS items were rated as “not at all present”, “present several days”, “present more than half of the days”, and “present nearly every day”. Symptoms were rated as positive whenever present “more than half of the days” or “nearly every day”. To make the instrument comparable to the Diagnostic and Statistical Manual of Mental Disorders Fourth/Fifth Edition (DSM-IV/V) criteria for GAD, the time criterion included the previous 6 months. The GADS has shown strong validity and reliability [19,28].

Formal assessment of DD was not available when FDS2 recruitment started in 2008. Although the PAID-20 was first developed in 1995 [29], evidence of its potential clinical utility appeared some years later [11]. The validity of an abbreviated 5-item Problem Areas in Diabetes Scale (PAID-5) questionnaire emerged around the time Year 6 FDS2 assessments commenced in 2014 [13]. The PAID-5 has since been confirmed as a robust self-administered instrument [18], and was incorporated into the suite of FDS2 questionnaires rather than its 20-item parent to reduce the time commitment and minimise duplication of the information collected. DD was diagnosed based on a PAID-5 score > 8 [18].

The 12-Item Short Form Health Survey version 2 (SF-12v2^®^), a self-reported measure of the impact of health on an individual’s everyday life [30], was also administered. The SF-12v2^®^ examines the domains of limitations in physical activities due to health problems, limitations in social activities due to physical or emotional problems, limitations in usual role activities due to physical health problems, bodily pain, general mental health (including psychological distress), and limitations in usual role activities due to physical health problems, vitality, and general health perceptions. We also included the 19-item Audit of Diabetes-Dependent Quality of Life (ADDQoL-19) [31], which consists of measurement of generic overall quality of life (QoL), and a further 19 items assessing the impact of diabetes on specific aspects of life such as leisure activities, working life, local or long-distance journeys, holidays, physical health, and family life. A weighted score for each domain was calculated as a multiplier of impact rating and importance, with lower scores reflecting worse QoL. A mean weighted impact score was calculated for the entire scale across all applicable domains.

### 2.3. Statistical Analysis

Stata Statistical Software Release 15 (StataCorp LLC, College Station, TX, USA) was used for LCA. LCA is a statistical method for evaluating internal construct validity through statistically modelling relationships implicit in the general concept of diagnostic criteria [32]. The latent classification provides a clinically optimal, external, construct-valid classification based on a given set of features with no a priori decision rules [33]. In the present study, LCA identified and created constructs from unobserved, or latent, subgroups based on individual responses to the PAID-5, PHQ-9, and GADS items. LCA assumes that a population of individuals is a mixture of distinct, but internally homogeneous subgroups [33]. LCA assesses the symptom profile of individuals and produces classes of individuals as suggested by their pattern of symptoms.

For the primary analysis, we included the five PAID-5 items, the nine DSM-IV/5 symptoms for major depression, and the items of worrying/anxiety, feeling restless, feeling tense, and feeling irritable from the GADS (the remaining excluded items overlapped with PHQ-9 items). All items were dichotomised (<2=0, ≥2=1) before LCA was performed. We estimated models with one to five classes, and each participant was assigned to the latent class to which the largest posterior probability was calculated. The best-fitting model was chosen based on the lowest value for the BIC. The likelihood ratio goodness-of-fit for the model versus the saturated model was determined. The normalised entropy of the classification model was calculated, where a value of 0 means there is no certainty about which individual is in which class and a value of 1 means certainty that the classes are strongly separated. We undertook two sensitivity analyses. The first included all nine GADS items in the LCA. The second included only Anglo-Celt participants (*n* = 393) to exclude the effects of culture or non-English speaking background on class membership.

The computer package IBM SPSS Statistics for Windows Version 25.0 (IBM Corp, Armonk, NY, USA) was used to examine PAID-5, GADS, and PHQ-9 items, and demographic and clinical characteristics across latent classes. Data are presented as proportions, mean ± standard deviation (SD), geometric mean (SD range), or median [interquartile range (IQR)] as appropriate. For two independent samples, we used Fisher’s exact test, for normally distributed variables, Student’s *t*-test, and for non-normally distributed variables, the Mann–Whitney U test. For multiple comparisons, we used Fisher’s exact test, or the chi-squared test for proportions. One-way ANOVA was applied for normally distributed variables, and we used the Kruskal–Wallis H-test for non-normally distributed variables. Bonferroni correction was used to adjust for multiple pairwise comparisons. Multinomial regression identified independent associates of LCA class membership. Plausible clinical variables with <5% missing data and *p* < 0.10 in bivariable analysis were considered for entry. A two-tailed significance level of *p* < 0.05 was used throughout.

## 3. Results

### 3.1. Baseline Characteristics

Compared with the 820 participants with type 2 diabetes recruited to FDS2 who either did not attend the Year 6 assessment or did not complete the PAID-5 questionnaire, the 662 included in the present study were significantly younger at baseline (mean ± SD 67.2 ± 12.4 vs. 64.0 ± 10.1 years, *p* < 0.001), more likely to be male (47.4% vs. 56.6%, *p* < 0.001), had a shorter median diabetes duration (10.0 [4.0–17.0] vs. 7.0 [2.0–14.6] years, *p* < 0.001), and were less likely to have GAD (6.1% vs. 3.1%, *p* = 0.010) or be depressed (16.1% vs. 8.8% subsyndromal depression (SSD) or major depression, *p* < 0.001; 7.7% vs. 4.3% major depression, *p* = 0.009).

### 3.2. Latent Class Analysis

**Figure 1:**
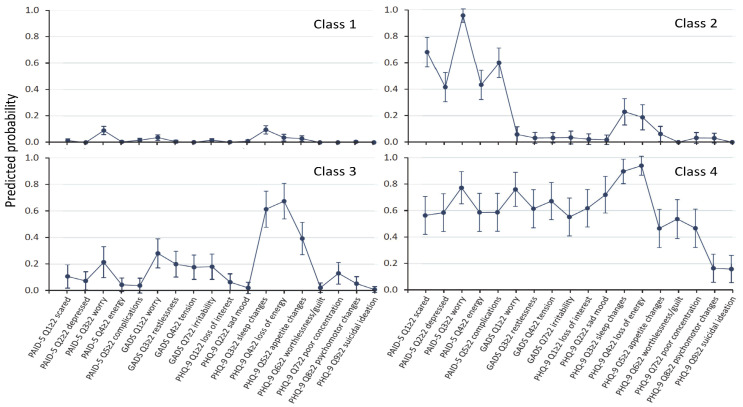
Symptom profile of partial conditional probabilities with 95% confidence intervals from latent class analysis for Class 1 (labelled “no symptoms”), Class 2 (“diabetes distress”), Class 3 (“subsyndromal depression”), and Class 4 (“major depression”). There were zero or near-zero predicted probabilities of each item in Class 1, high predicted probabilities (>0.4) of diabetes distress items but zero predicted probabilities of non-overlapping generalised anxiety disorder or depressive symptoms in Class 2, high predicted probabilities for sleep changes, loss of energy, and appetite changes in Class 3, and high predicted probabilities for all items except psychomotor changes and suicidal ideation in Class 4.

A four-class LCA model was identified as superior based on the Bayesian information criterion (BIC; all goodness-of-fit *p* > 0.999, suggesting saturation for all models). For this model, posterior probabilities were 0.969 for the largest Class 1 (*n* = 435; 65.7%), 0.927 for Class 2 (*n* = 93; 14.0%), 0.875 for Class 3 (*n* = 84; 12.6%), and 0.967 for Class 4 (*n* = 50; 7.5%). Entropy was 0.895. The predicted probabilities of each item for each of the four classes for the whole sample are shown in Figure 1.

**Figure 2:**
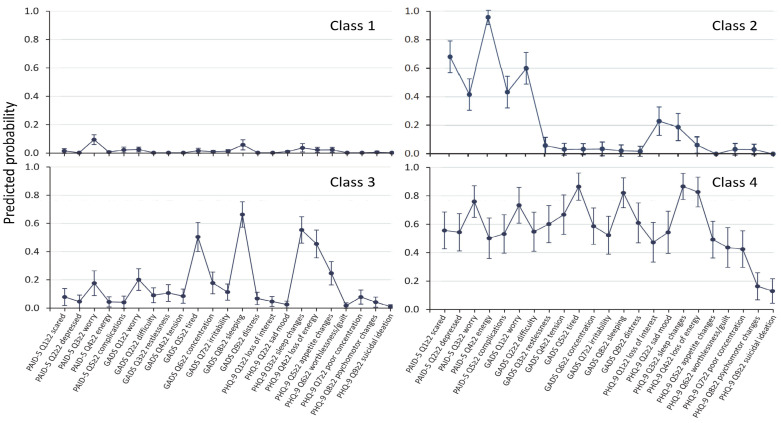
Symptom profile of partial conditional probabilities with 95% confidence intervals from latent class analysis for four classes with all nine GADS items included. The only difference from Figure 1 was that the SSD group also included the GADS items of feeling tired and difficulty sleeping.

**Figure 3:**
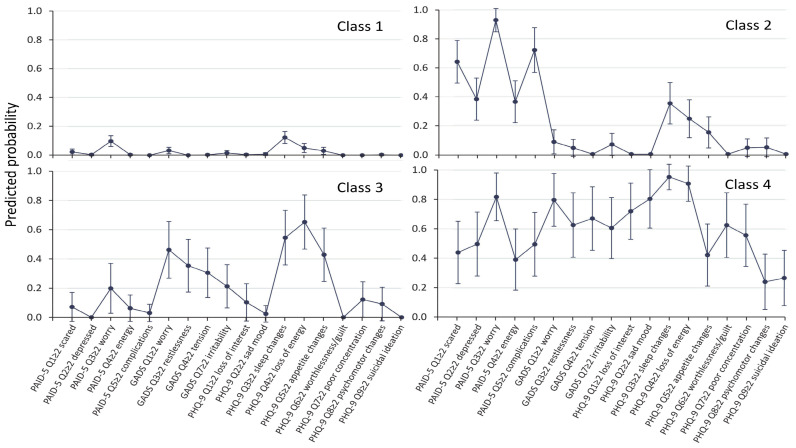
Symptom profile of partial conditional probabilities with 95% confidence intervals from latent class analysis for four classes with Anglo-Celt participants included. The findings were similar to those in Figure 1.

Class 1 (“no symptoms”) has zero or near-zero predicted probabilities of each item. Class 2 (DD) has high predicted probabilities (>0.4) for all five DD items, but low probabilities (<0.4) for any GAD or depression symptoms. Class 3 (SSD) has high predicted probabilities for sleep and appetite changes and loss of energy, but low (<0.4) or close to zero probabilities for the remaining items. Class 4 (“major depression” (MD)) has high predicted probabilities for all five PAID items, all four GADS items, and all depression items except for psychomotor changes and suicide ideation. When the LCA was repeated with all nine GADS items included, the only difference was that the SSD group also included the GADS items of feeling tired and difficulty sleeping (see Figure 2), and when LCA was performed only for the Anglo-Celts, the findings remained unchanged (see Figure 3).

### 3.3. Mean Scores for Diabetes Distress, Anxiety, and Depressive Items for the Four LCA Classes

The mean scores for each of the 18 items by LCA class are shown in Table 1. Scores on the PAID-5 showed that (i) the DD class scored significantly higher than “no symptoms” and the SSD classes on all PAID-5 items, (ii) the MD class scored significantly higher on the PAID-5 depression and energy items than the DD group (with no significant between-class differences for the remaining PAID-5 items), and it showed significantly higher scores on all PAID-5 items compared to the SSD and “no symptoms” classes.

For the GADS data, (i) individuals in the DD class showed significantly higher scores on the worry item compared to the “no symptoms” class, (ii) the SSD class showed significantly higher scores on the GADS items of worry, restlessness, and tension compared to the “no symptoms” class, and (iii) the MD class showed significantly higher scores on all GADS items compared to the other three classes.

The PHQ-9 data showed that (i) the DD class had significantly higher scores on the PHQ-9 items of loss of interest, sad mood, sleep changes, loss of energy, and appetite and psychomotor changes compared to the “no symptoms” class, (ii) the SSD class showed significantly higher scores on all the PHQ-9 items except for suicidal ideation compared to the “no symptoms” class, and significantly higher scores on the items of sleep and appetite changes, loss of energy, and poor concentration compared to the DD class, and (iii) the MD class showed significantly higher scores on all the PHQ-9 items compared to the “no symptoms” and DD classes, and on all PHQ-9 items except for appetite changes compared to the SSD class.

### 3.4. Characteristics at Year 6 across LCA Classes

Bivariable analyses of clinical and psychiatric characteristics at Year 6 by class are summarised in Table 2. Both the DD and the MD classes showed a similar DD prevalence, which was significantly higher compared to the “no symptoms” and SSD classes. The MD class showed a significantly higher prevalence of SSD, MD, and GAD than the other three classes. The DD and SSD classes showed significantly lower scores on the 12-Item Short Form Health Survey version 2 (SF-12v2^®^) mental and physical components compared to the “no symptoms” class, whereas the MD class showed significantly lower mental component scores compared to the other three classes, and significantly lower physical component scores compared to the “no symptoms” and DD classes. The overall 19-item Audit of Diabetes-Dependent Quality of Life (ADDQoL-19) scores were significantly lower for the DD and SSD classes compared to the “no symptoms” class, whereas the MD class showed significantly lower scores compared to all three other classes. The impact of diabetes on quality of life was significantly worse for those in the DD and MD classes compared to the “no symptoms” or SSD classes.

In relation to key clinical features, both the DD and SSD classes showed significantly higher HbA_1c_ versus the “no symptoms” class. BMI was significantly higher for the DD and MD classes compared to the “no symptoms” and SSD classes. However, there were no significant between-class differences in fasting serum glucose, ABSI, central obesity by waist circumference, heart rate, systolic and diastolic blood pressure, antihypertensive medication use, total serum cholesterol, lipid-modifying medication use, aspirin use, urinary albumin:creatinine ratio, retinopathy, peripheral sensory neuropathy, or peripheral arterial disease. Importantly, there were no significant between-class differences in SMBG or diabetes medication compliance.

### 3.5. Multinomial Regression Model of Independent Associates of LCA Classes at Year 6

A multinomial regression model analysis using the “no symptoms” class as a reference showed a significant association between the DD class and HbA_1c_, BMI, and Southern European and Asian ethnic backgrounds (Table 3). The SSD class was significantly associated with HbA_1c_ and cerebrovascular or coronary heart disease. The MD class had significant associations with age (negatively), HbA_1c_, BMI, Southern European ethnic background, and coronary heart disease.

## 4. Discussion

We examined the syndromic validity of DD and its overlap with anxiety and depression. Our first hypothesis, that DD constitutes an independent psychological class, was substantiated by LCA, which demonstrated a single class representing 14.0% of the sample, with high scores for all five PAID-5 items. Our second hypothesis was not substantiated since there were no between-class differences in diabetes medication and SMBG adherence. Our third hypothesis of a phenomenological overlap between DD, depression, and anxiety was statistically substantiated. Importantly, we could not confirm the relevance of DD as a marker of adverse diabetes status since membership of the MD and SSD classes had stronger clinical implications than the DD class in terms of higher prevalence of cerebrovascular and coronary heart disease. The MD class had the worst health status of all four classes. The SSD and DD classes had similar overall health status, which was worse than for those with no symptoms. Nonetheless, diabetes adversely impacted quality of life similarly in the MD and DD classes.

To our knowledge, this is the first study to use LCA to examine the phenomenology and nosological position of DD. One in seven of our participants were in a class that loaded on all PAID-5 items but on none of the items for depression or anxiety. An important psychometric limitation of the PAID-5 is the lack of validation against a gold-standard criterion, and there are no diagnostic criteria (or even a standardised concept) for DD. The criterion for diagnosing DD using the PAID-5 is a cut-off score using the PAID-20 as reference [11]. Our LCA identified three other classes, one containing most participants who had no emotional/psychological disorders (65.7%), one with SSD (12.7%), and a class with MD, anxiety, and DD (7.6%). This latter class showed a 56% prevalence of MD compared to <1% for the other three classes, as well as a higher prevalence of GAD (40%) compared to <4% for the other three classes.

As well as a lack of important psychiatric correlates in the DD group, there were relatively weak independent associations between DD and relevant demographic and clinical aspects of diabetes. A multinomial regression model of independent associates of LCA classes defined by DD, GAD, and depression items with reference to the “no symptoms” class showed a significant association between the MD class and age (negatively), Southern European ethnicity, BMI, HbA_1c_, and coronary heart disease. The SSD class showed a significant association with HbA_1c_ and cerebrovascular and coronary heart disease. DD was also associated with Southern European ethnic background, HbA_1c_, and BMI, with Asian ethnic background the only variable uniquely related to DD class membership. An increased prevalence of DD has been reported previously in people with type 2 diabetes in ethnic minorities in developed countries [34].

DD has been identified as a significant predictor of complications [35] but there is a more extensive literature demonstrating depression as a correlate and predictor of diabetes-associated morbidity and mortality. Depression has been associated with increased micro- and macrovascular complications, and increased mortality [36,37]. The association between depression and worse diabetes self-care is present for both MD and SSD. Gonzalez et al. found that depression remained associated with diabetes self-care outcomes such as poor adherence to diet, less frequent exercise, and non-adherence to prescribed medication after adjusting for PAID-20 scores [38]. Depression is associated with poor glycaemic control, a greater number of health complications, worse quality of life, increased functional impairment, more hospital days and days off work, increased healthcare use and costs, reduced diabetes treatment adherence and self-management, and increased mortality [14,39,40,41,42,43]. Moreover, depression in type 2 diabetes is often unrecognised and untreated [36], and it has yet to be demonstrated that screening for DD may help to diagnose MD.

The present study had some limitations. First, our sample comprised 662 participants assessed for DD six years after baseline. The 820 individuals who did not have a Year 6 DD assessment were significantly older, more likely female, had longer a diabetes duration, and had a higher prevalence of GAD and MD at baseline. Nevertheless, our study showed a prevalence of MD and GAD consistent with previous reports [7] and has a relatively large sample size in comparison to other DD studies [6]. Second, this is a cross-sectional study and longitudinal outcome studies are required to further examine the clinical validity of the classes we identified. Third, given the burden of assessments including multiple and potentially overlapping questionnaires, participants were not assessed with structured psychiatric interviews for depression and anxiety. However, we assessed the severity of depression and anxiety using simple instruments with strong reliability and validity in diabetes [19]. Finally, whereas we used the PAID-5 rather than the 20-item PAID version, several studies have demonstrated that scores from both versions correlate strongly [11,18].

## 5. Conclusions

Based on our findings, DD may have limited clinical utility. We found an independent LCA-defined class of DD in community-based Australians with type 2 diabetes, which showed few relevant clinical correlates compared to the SSD or MD classes. MD was associated with the worst health status and outcomes, whereas DD had no clinically relevant independent correlates. Therefore, a more efficient approach to capture the emotional problems associated with type 2 diabetes and associated increased morbidity is to screen for depression and its severity as well as for syndromal anxiety rather than DD.

## Figures and Tables

**Table 1:** Mean scores ± SD for diabetes distress, anxiety, and depressive items for the four LCA classes.

Item	Class 1: No Symptoms	Class 2: Diabetes Distress	Class 3: Subsyndromal Depression	Class 4: Major Depression	F	ANOVA *p*-Value
N (%)	435 (65.7)	93 (14.0)	84 (12.7)	50 (7.6)		
PAID-5: Scared	0.28 ± 0.47	1.92 ± 1.08 ***	0.46 ± 0.75 ^†††^	1.64 ± 1.23 ***^,‡‡‡^	178.7	<0.001
Depressed	0.16 ± 0.36	1.31 ± 0.88 ***	0.37 ± 0.66 *^,†††^	1.76 ± 1.26 ***^,†††,‡‡‡^	174.7	<0.001
Worry	0.63 ± 0.72	2.43 ± 0.67 ***	0.80 ± 0.88 ^†††^	2.46 ± 1.22 ***^,‡‡‡^	195.6	<0.001
Energy	0.14 ± 0.36	1.28 ± 0.99 ***	0.30 ± 0.53 ^†††^	1.80 ± 1.14 ***^,†††,‡‡‡^	185.8	<0.001
Complications	0.28 ± 0.48	1.73 ± 1.01 ***	0.40 ± 0.58 ^†††^	1.90 ± 1.22 ***^,‡‡‡^	187.9	<0.001
GADS: Worry	0.43 ± 0.58	0.71 ± 0.64 **	1.05 ± 0.82 ***^,††^	2.10 ± 0.93 ***^,†††,‡‡‡^	107.6	<0.001
Restlessness	0.31 ± 0.47	0.46 ± 0.56	0.80 ± 0.86 ***^,††^	1.62 ± 0.86 ***^,†††,‡‡‡^	84.8	<0.001
Tension	0.34 ± 0.48	0.48 ± 0.56	0.77 ± 0.77 ***^,††^	1.72 ± 0.88 ***^, †††,‡‡‡^	94.2	<0.001
Irritability	0.39 ± 0.52	0.55 ± 0.56	0.79 ± 0.84 ***	1.56 ± 0.99 ***^,†††,‡‡‡^	57.5	<0.001
PHQ-9: Loss of interest	0.16 ± 0.37	0.45 ± 0.58 ***	0.57 ± 0.77 ***	1.74 ± 1.01 ***^,†††,‡‡‡^	136.9	<0.001
Sad mood	0.17 ± 0.40	0.41 ± 0.54 ***	0.38 ± 0.54 **	1.88 ± 0.92 ***^,†††,‡‡‡^	180.4	<0.001
Sleep changes	0.56 ± 0.79	0.97 ± 0.91 ***	1.76 ± 1.10 ***^,†††^	2.40 ± 0.78 ***^,†††,‡‡‡^	103.4	<0.001
Loss of energy	0.55 ± 0.61	0.98 ± 0.82 ***	2.01 ± 0.83 ***^,†††^	2.48 ± 0.61 ***^,†††,‡‡^	204.6	<0.001
Appetite changes	0.23 ± 0.49	0.52 ± 0.69 **	1.18 ± 1.06 ***^,†††^	1.36 ± 1.12 ***^,†††^	78.2	<0.001
Worthlessness/guilt	0.08 ± 0.27	0.27 ± 0.45 **	0.33 ± 0.52 ***	1.48 ± 1.09 ***^,†††,‡‡‡^	150.9	<0.001
Poor concentration	0.12 ± 0.32	0.28 ± 0.56	0.55 ± 0.88 ***^,††^	1.40 ± 1.09 ***^,†††,‡‡‡^	89.5	<0.001
Psychomotor changes	0.04 ± 0.26	0.24 ± 0.54 ***	0.26 ± 0.64 ***	0.68 ± 0.79 ***^,†††,‡‡‡^	38.5	<0.001
Suicide ideation	0.02 ± 0.13	0.06 ± 0.25	0.11 ± 0.41	0.58 ± 0.81 ***^,†††,‡‡‡^	53.6	<0.001

Pairwise comparisons with Bonferroni correction for multiple comparisons: * *p* < 0.05, ** *p <* 0.01, *** *p <* 0.001 vs. Class 1; ^††^ *p <* 0.01, ^†††^ *p <* 0.001 vs. Class 2; ^‡‡‡^ *p <* 0.001 vs. Class 3.

**Table 2:** Characteristics across LCA classes defined by diabetes distress items and symptoms of depression and generalised anxiety disorder at Year 6 for the 662 participants with type 2 diabetes.

	Missing (*n*)	Class 1: No Symptoms	Class 2: Diabetes Distress	Class 3: Subsyndromal Depression	Class 4: Major Depression	Trend *p*-Value
Predicted posterior probability	0	0.969 ± 0.061	0.927 ± 0.106	0.875 ± 0.144	0.967 ± 0.079	
PAID-5 score ≥ 8 (%)		0.2	61.3 ***	3.6 ^†††^	64.0 ***^,‡‡‡^	<0.001
Major or minor depressive syndrome (%)	0	0	3.2 *	8.3 ***	82.0 ***^,†††,‡‡‡^	<0.001
Major depressive syndrome (%)	0	0	0	1.2	56.0 ***^,†††,‡‡‡^	<0.001
Generalised anxiety disorder (%)	0	0	0	3.6 *	40.0 ***^,†††,‡‡‡^	<0.001
Antidepressant ± anti-anxiety medication (%)	14	13.2	16.1	24.4	30.0 *	0.004
SF-12 v2:	9					
Mental component score (%)		57 [52–60]	50 [44–58] ***	47 [39–53] ***	38 [29–44] ***^,†††,‡‡‡^	<0.001
Physical component score (%)		46 [38–53]	39 [32–48] ***	38 [28–48] ***	31 [24–41] ***^,††^	<0.001
ADDQoL scores:						
Overall quality of life (*n* = 623)	39	2 [1, 2]	1 [1, 2] ***	1 [0, 2] ***	0 [0, 1] ***^,†††,‡‡‡^	<0.001
If person did not have diabetes (*n* = 623)	39	−1 [−1, 0]	−2 [−2, −1] ***	−1 [−2, 0] ^†††^	−2 [−3, −1] ***^,‡‡^	<0.001
Weighted sum (*n* = 611)	51	−0.4 [−1.1, −0.1]	−1.3 [−3.3, −0.6] ***	−0.7 [−2.0, −0.3] **^,†††^	−2.0 [−3.9, −0.4] ***^,‡‡‡^	<0.001
Age (years)	0	71.3 ± 10.2	69.0 ± 9.6	70.3 ± 9.2	66.0 ± 10.0 **	0.002
Male (%)	0	56.8	58.1	58.3	50.0	0.782
Education level attained (%):	6					0.406
Primary or less		6.7	5.4	8.4	14.3	
Secondary		50.3	50.5	56.6	49.0	
Tertiary		42.9	44.1	34.9	36.7	
Not fluent in English (%)	0	4.1	6.5	7.1	16.0 *	0.012
Ethnic background (%):	0		*		*	0.002
Anglo-Celt		64.6	47.3	53.6	46.0	
Southern European		7.4	16.1	13.1	18.0	
Other European		6.7	8.6	6.0	16.0	
Asian		3.9	9.7	<6.0 ^b^	<10.0 ^b^	
Aboriginal		1.6	<5.4 ^b^	<6.0 ^b^	<10.0 ^b^	
Mixed/other		15.9	16.1	23.8	12.0	
Current smoker (%)	1	4.4	<5.4 ^b^	<6.0 ^b^	10.2	0.189
Alcohol (standard drinks/day)	22	0.3 [0–1.2]	0.1 [0–0.3] *	0.1 [0–0.8]	0.1 [0–0.3]	0.001
Age at diabetes diagnosis (years)	0	56.5 ± 10.9	52.4 ± 10.6 **	54.3 ± 11.6	49.6 ± 9.4 ***	<0.001
Diabetes duration (years)	0	12.7 [8.0–20.5]	15.5 [8.3–21.8]	14.4 [8.4–21.0]	14.8 [10.4–22.7]	0.203
Diabetes treatment (%):	5				*	0.003
Diet/exercise		20.6	7.7	10.7	10.2	
OGLMs or non-insulin injectable		54.3	57.1	57.1	40.8	
Insulin only		6.0	9.9	10.7	10.2	
Insulin + OGLMs or non-insulin injectables		19.2	25.3	21.4	38.8	
Fully compliant with diabetes medication ^a^ (%)		74.6	77.8	72.6	68.3	0.692
Self-monitors blood glucose (%)	11	72.4	77.8	67.1	79.2	0.333
Fasting serum glucose (mmol/L)	1	7.6 [6.4–9.0]	8.2 [6.3–10.6]	7.9 [6.1–9.9]	8.1 [6.8–10.2]	0.303
HbA_1c_ (mmol/mol)	1	51 [44–60]	60 [51–68] ***	57 [45–68] *	55 [46–74]	<0.001
HbA_1c_ (%)	1	6.8 [6.2–7.6]	7.6 [6.8–8.4] ***	7.4 [6.3–8.4] *	7.2 [6.4–8.9]	<0.001
A Body Shape Index (m^11/6^ /kg^2/3^)	6	0.081 ± 0.005	0.081 ± 0.004	0.082 ± 0.005	0.081 ± 0.005	0.224
BMI (kg/m^2^)	5	30.5 ± 5.8	33.0 ± 5.9 **	31.9 ± 5.9	33.5 ± 7.1 **	<0.001
Central obesity (% by waist circumference)	5	66.2	79.3	74.7	76.0	0.038
Heart rate (beats per minute)	1	69 ± 11	72 ± 11	71 ± 12	71 ± 13	0.061
Systolic blood pressure (mmHg)	1	139 ± 19	141 ± 20	140 ± 20	139 ± 21	0.911
Diastolic blood pressure (mmHg)	1	77 ± 12	77 ± 11	79 ± 11	77 ± 13	0.535
On antihypertensive medication (%)	13	80.4	84.9	91.6	84.0	0.078
Total serum cholesterol (mmol/L)	1	4.3 ± 1.1	4.1 ± 0.9	4.2 ± 1.2	4.2 ± 1.2	0.488
HDL cholesterol (mmol/L)	1	1.20 ± 0.33	1.13 ± 0.33	1.13 ± 0.31	1.07 ± 0.28 *	0.007
Serum triglycerides (mmol/L)	1	1.5 (0.9–2.4)	1.6 (0.9–2.6)	1.7 (1.1–2.7)	1.7 (0.9–3.0)	0.076
On lipid-modifying medication (%)	13	77.3	75.3	79.5	74.0	0.856
On aspirin (%)	13	35.2	45.2	42.2	44.0	0.184
Urinary albumin:creatinine ratio (mg/mmol)	7	2.6 (0.6–11.6)	3.6 (0.7–19.5)	2.3 (0.6–8.9)	3.5 (0.6–21.4)	0.136
eGFR (CKD-EPI) (mL/min/1.73 m^2^)	1	74 ± 19	74 ± 21	69 ± 21	76 ± 21	0.168
Any retinopathy (%)	69	13.2	15.7	19.2	23.3	0.205
Peripheral sensory neuropathy (%)	0	54.5	50.5	65.5	58.0	0.201
Peripheral arterial disease (%)	10	9.3	8.7	11.3	16.7	0.376
Coronary heart disease (%)	0	28.0	29.0	45.2 *	44.0	0.004
Cerebrovascular disease (%)	0	4.8	10.8	15.5 **	12.0	0.002

Data are presented as percentage (%), mean ± standard deviation (SD), median [interquartile range], or geometric mean (SD range). Multiple comparisons for proportions were calculated with Fisher’s exact test or chi-squared test, for normally distributed variables, with one-way ANOVA, and for non-normally distributed variables, with Kruskal–Wallis H test. Pairwise comparisons with Bonferroni correction for multiple comparisons: * *p* < 0.05, ** *p* < 0.01, *** *p* < 0.001 vs. Class 1; ^††^ *p* < 0.01, ^†††^ *p* < 0.001 vs. Class 2; ^‡‡^ *p* < 0.01, ^‡‡‡^ *p* < 0.001 vs. Class 3; OGLM = oral glucose-lowering medication; ^a^ for those not treated with diet and exercise alone. ^b^ Exact numbers withheld to ensure participant privacy and confidentiality.

**Table 3:** Multinomial regression model of independent associates of LCA classes defined by diabetes distress items and symptoms of depression and generalised anxiety disorder at Year 6, with reference to the largest class with no symptoms (Class 1), for the 662 participants with type 2 diabetes.

		Odds Ratio (95% CI)	
	Class 2: Diabetes Distress	Class 3: Subsyndromal Depression	Class 4: Major Depression
Age (increase of 10 years)	0.94 (0.73, 1.21)	0.87 (0.67, 1.13)	0.59 (0.42, 0.81)
Southern European	2.37 (1.19, 4.72)	1.73 (0.81, 3.67)	2.43 (1.04, 5.69)
Asian	3.68 (1.42, 9.58)	0.36 (0.05, 2.82)	1.48 (0.31, 7.10)
BMI at Year 6 (increase of 5 kg/m^2^)	1.39 (1.14, 1.70)	1.12 (0.90, 1.38)	1.30 (1.01, 1.67)
HbA_1c_ at Year 6 (increase of 11 mmol/mol or 1%)	1.36 (1.14, 1.63)	1.35 (1.13, 1.62)	1.27 (1.02, 1.60)
History of cerebrovascular disease	2.23 (0.96, 5.20)	2.86 (1.29, 6.32)	2.51 (0.90, 7.02)
History of coronary heart disease	1.08 (0.63, 1.87)	2.16 (1.29, 3.63)	2.91 (1.49, 5.66)

## Data Availability

Some outcome data supporting the findings of this study are available from the Western Australian Department of Health, but restrictions apply to the availability of these data, which were used under strict conditions of confidentiality for the current study, and so are not publicly available. Data are, however, available from the authors upon reasonable request and with permission of the Western Australian Department of Health.
